# Treating the injured: a privilege conferred by both patient and wider society: the medical exception and consent in trauma

**DOI:** 10.1302/2633-1462.57.BJO-2024-0052

**Published:** 2024-07-09

**Authors:** Simon Britten

**Affiliations:** 1 Leeds Teaching Hospitals, Leeds, UK

**Keywords:** The medical exception, Proper medical treatment, Reasonable treatment options, *Montgomery* 2015, *McCulloch* 2023, Offences against the Person Act 1861, Civil law, Criminal law, Consent, Consent in trauma, Orthopaedic Trauma, Orthopaedic Surgeons, Physicians, orthopaedic trauma surgeons, orthopaedic trauma surgeons, limb injury, wounding, Limb Reconstruction, surgical treatment, alternative treatments

## Abstract

Two discrete legal factors enable the surgeon to treat an injured patient the fully informed, autonomous consent of the adult patient with capacity via civil law; and the medical exception to the criminal law. This article discusses current concepts in consent in trauma; and also considers the perhaps less well known medical exception to the Offences against the Person Act 1861, which exempts surgeons from criminal liability as long as they provide ‘proper medical treatment’.

Cite this article: *Bone Jt Open* 2024;5(7):565–569.

## Introduction

If, in a seemingly random and brutal act, an individual amputates the limb of a total stranger, this would potentially leave the individual wide open to criminal charges of wounding with intent to cause grievous bodily harm, and a civil claim under the tort of battery. As orthopaedic trauma surgeons, we may find ourselves carrying out exactly such an act, and drilling into limbs, and repeatedly striking with a hammer, and excising joints, and so on… yet not only are we permitted to do so, we are, in the right circumstances, encouraged and paid to do so. This is on the tacit understanding that the recipient of our actions will, in the longer term, be better off in terms of symptoms, function, and quality of life than if we have not carried out such acts. It has always struck me as a rather incongruous way to care for our fellow humans, but that is what orthopaedic surgeons do.

With all the discussion and hype surrounding the issue of informed consent in the years since the *Montgomery* judgment of the UK Supreme Court in 2015,^1^ one could be forgiven for believing that seeking and receiving the fully informed, autonomous consent of the adult patient with capacity was the only criterion necessary to go ahead with an orthopaedic trauma operation. But this is not so. Before the individual patient can begin to weigh up their options for any proposed surgery, wider society, via the law, has already set the scene to permit and enable the surgeon to carry out ‘proper medical treatment’.

## The medical exception

Given the potentially very invasive nature of medicine, and in particular surgery, it became apparent during Victorian times of the necessity to take medical procedures outside the remit of criminal law. Without placing medical and surgical treatment outside the criminal law, some of the more invasive activities of physicians and surgeons would fall within the scope of the Offences against the Person Act 1861,^2^ rendering such activities potentially serially criminal. However, as long as the surgeon is considered to be providing ‘proper medical treatment’, then their actions stand outside the criminal law. This is the medical exception to the Offences against the Person Act 1861, making surgeons’ actions exempt from the usual parameters of criminal liability; consent alone is not enough.

In the non-medical and rather curious case of *R v Brown*,^3^ involving consensual sadomasochistic practices over several years between a group of homosexual men, in which no one required any medical treatment for any injury, the House of Lords reiterated that consent alone is insufficient to make inflicting actual bodily harm and wounding lawful. This was on the basis that it was “not in the public interest that people should try to cause or should cause each other actual bodily harm for no good reason.” In order to distinguish such non-medical acts from the medical, Lord Mustill noted that “much of the bodily invasion involved in surgery lies well above the point at which consent could even arguably be regarded as furnishing a defence. Why is this so? The answer must in my opinion be that proper medical treatment, for which actual or deemed consent is a prerequisite, is in a category of its own.”^4^ He also emphasized the importance of invasive surgical treatment being “performed in accordance with good medical practice…”^5^

Lord Mustill also sat in the case of *Bland*.^6^ Tony Bland was the 96th Hillsborough disaster victim, who died after almost four years in a persistent vegetative state. In *Bland*, Lord Mustill stated that “bodily invasions in the course of proper medical treatment stand completely outside the criminal law” when in general discourse referencing the hypothetical surgical amputation of a diseased hand.^7^ Back in 1925, Lord Atkin had stated that “the mere consent of the patient to an operation which may be considered contrary to public policy, or likely to be an injury to the public generally, is not such an operation as would relieve the surgeon from [criminal] responsibility.”^8^ In the mid-1990s when the Law Commission considered the interplay between consent and criminal law, the recognition of proper medical treatment as the determinant of the medical exception “…turns on other matters unrelated to consent…”, with emphasis given to the purpose of the surgery proposed, and it being in the public interest to undertake such a procedure.^9^ Furthermore, the Commission proposed that the medical exception should cover only the activities of those who are medically qualified, distinguishing between “those whom [the Commission] trusts to engage in the consensual infliction of harm and those who are not to be trusted.”^10^

Over centuries, both governments and medics have attempted to set out who can do what to whom while administering medical treatment. In those attempts, two things were constant – the physicians always believed that they alone were capable of delivering proper medical treatment, and the ‘quack’ was always somebody else. In 1421, a physicians’ petition was presented to Parliament requesting “that no man, of no manner, estate, degree, or condition, practise in Physic, from this time forward, but he have long time used the Schools of Physic within some University, and be graduated in the same…”. They also requested that no woman be allowed to practise Physic on pain of long imprisonment and paying £40 to the King.^11^ The Physicians and Surgeons Act 1511 brought medical practitioners under the jurisdiction of the relevant bishop of each local diocese, aiming to allow only those who had been examined to practise medicine.^12^ In 1518, the Royal College of Physicians was founded by Royal Charter, and the regulation of those entitled to practise medicine was established, with a hierarchy of physicians, then surgeons, and then apothecaries. In 1540, the Fellowship of Surgeons merged with the Company of Barbers to form the Company of Barber-Surgeons,^13^ and by 1800 the Royal College of Surgeons of London (later England) was formed by Royal Charter.^14^ While licensed practitioners were jostling for a place in the historical healthcare marketplace, all manner of unlicensed individuals also set out their stalls. Until around the middle of the 19th century, one unintentional irony is that wealthy individuals who had purchased their medical academic qualifications could potentially do much more harm with their rather invasive but unscientific methods than the homeopathist, whose diluted potions would make no one better, but at the same time did little or no further harm.

Kennedy considered that surgery may be considered lawful if performed by “experienced practitioners” and provided that “there is at least some risk of harm to the patient if surgery is not performed.”^15^ The bioethicists’ distillate of what constitutes proper medical treatment can be summarized as a procedure performed by someone appropriately qualified, with a purpose that is beneficent and in the wider public interest, designed with intention to benefit the patient, with a good reason to do it, and the proposed treatment considered a reasonable thing to do.^16^

## Surgeons and the criminal law

During their careers, surgeons may encounter various and disparate sources of professional jeopardy. Hopefully infrequent brushes with the law are confined to civil matters – being sued for clinical negligence, disciplinary trouble with the employing Trust, referral to the General Medical Council (GMC) and a fitness-to-practise hearing with the Medical Practitioners Tribunal Service, investigation by the Parliamentary and Health Service Ombudsman for an unresolved patient complaint, or a trip to Coroner’s court if things have gone really badly. It is rare for surgeons, in the course of their professional activities, to find themselves on the wrong side of the criminal law, particularly as “a well-intentioned doctor should not be treated as a common criminal.”^17^

One interface between the criminal law and medicine is that of gross negligence manslaughter, such as the case of *R v Sellu*, in which surgeon David Sellu was sentenced to two and a half years in prison, of which he served 15 months, before his conviction was subsequently quashed on appeal.^18^ While another recent criminal case of breast surgeon Ian Paterson is well known, even notorious, the details of his conviction for 17 counts of wounding with intent and three counts of unlawful wounding, contrary to the Offences against the Person Act 1861, are more opaque.^19^ While much was made in the media of Paterson’s non-standard 'cleavage-sparing mastectomy', he was sentenced to 15 years in prison, increased to 20 years on appeal, for something else entirely. The case of *Paterson* considered “a somewhat novel issue, not previously determined by the courts, as to whether an individual can rely on [the] medical exception in circumstances where patients are not told the true facts about their medical condition, where the medical procedure was not for a proper medical purpose, and where the doctor concerned knew this to be the case.”^20^

The sentencing remarks at Paterson’s original trial state that in patients with little or no risk of developing breast cancer, following initial investigations, he deliberately exaggerated the risk of cancer, then arranged unnecessary surveillance and in some cases unnecessary surgical treatment, including mastectomies and reconstruction, in patients rendered understandably anxious and vulnerable, leading to subsequent profound physical and psychological harm.^21^ Mr Justice Baker continued: “…the offences of which you have been convicted…are not ones involving either negligence or even recklessness, where someone causes harm by either oversight, or knowingly or otherwise is working beyond their capabilities. On the contrary, as the jury found, these offences represent the intentional application of permanent harm by you upon patients who were in your care, for your own selfish purposes, rather than because they were necessary to maintain their health. In these circumstances, they represent the antithesis of the Hippocratic oath.”^21^

In *Paterson*, patients were misled as to the true nature of their condition, and this intentional misrepresentation prompted them, fearful for their health, to undergo surgery which the surgeon secretly knew was not for any proper medical purpose. In the face of such egregious deception, the surgery performed did not amount to proper medical treatment, and so there could be no medical exception from the Offences against the Person Act. Furthermore, the deception vitiated any notion of fully informed, autonomous consent to surgery. Such an extreme and unique case demonstrates the power of the medical exception in keeping decent surgeons away from serious aspects of criminal law. It also demonstrates that the medical exception and consent are discrete and yet interrelated factors in enabling us, as surgeons, to perform surgery at all.

## Consent in trauma

In November 2020, and in the wake of the *Montgomery* judgment,^1^ the GMC published updated guidance on informed consent.^22^ In a 40-page document containing 96 paragraphs, just three paragraphs were dedicated to the issue of treatment in emergencies, as follows:

- “In an emergency, decisions may have to be made quickly so there’ll be less time to apply this guidance in detail, but the principles remain the same. You must presume a conscious patient has capacity to make decisions and seek consent before providing treatment or care.
- In an emergency, if a patient is unconscious or you otherwise conclude that they lack capacity and it’s not possible to find out their wishes, you can provide treatment that is immediately necessary to save their life or prevent a serious deterioration of their condition. If there is more than one option, the treatment you provide should be the least restrictive of the patient’s rights and freedoms, including their future choices.
- For as long as the patient lacks capacity, you should provide ongoing care following the guidance in paragraphs 87 to 91. If the patient regains capacity while in your care, you must tell them what has been done and why, as soon as they are sufficiently recovered to understand. And you must discuss with them the options for any ongoing treatment.”^22^

When the guidance came out, the British Orthopaedic Association (BOA) Medico-legal Committee put together a group to discuss it, including representation from the BOA Trauma Group, Orthopaedic Trauma Society, and British Limb Reconstruction Society: senior orthopaedic trauma surgeons, senior counsel, a senior defence solicitor, and a GMC representative. Was the guidance too scant for the emergency trauma scenario, and was additional guidance needed? During another COVID-19 lockdown, could we meet virtually to create additional guidance? But a wise voice prevailed in the form of senior defence solicitor Bertie Leigh:^23^ “Be careful what you wish for!” With only three paragraphs to satisfy, it is a relatively low bar to clear.

Judges are intelligent people; it is reasonable to imagine their reaction when confronted with a common emergency scenario in which the consent process is queried: ‘It was a life-/limb-threatening emergency, best efforts were made to involve the patient in decision making, but in reality action was required immediately. Temporary fixation and vascular repair was performed, with a future discussion to be had with the patient once they had recovered sufficiently regarding amputation versus definitive attempts at reconstruction.’ A judge can understand this chain of events and actions. No additional guidance was produced. The bar remains relatively low in the true emergency, and is easier to clear than a long list of dos and don’ts.

While it is fairly obvious that the unconscious patient should be treated in their best interests as per GMC guidance, an additional consideration has been raised by a senior UK orthopaedic trauma surgeon. In 2016, Peter Worlock advocated that in cases of isolated severe limb injury, serious thought should be given to whether a patient can truly have capacity to consent, given severe pain and high doses of opiate analgesia,^24^ to which this author would add the psychological distress of literally having just been hit by a bus, for example. In such circumstances, despite the patient remaining conscious and with no suggestion of permanent cognitive impairment, the treating surgeon should be prepared to treat in best interests, with very careful documentation of how the decision was reached, using an NHS Consent form 4.^25^ This approach, when sometimes necessary, has been echoed anecdotally by another senior UK orthopaedic trauma surgeon, who described a case of severe limb trauma in a conscious patient admitted on his take, and as he began to (succinctly and swiftly) set out options for treatment, the patient exhorted, “just fix my bloody leg Professor!”^26^

Aside from limb trauma in extremis, in most circumstances the treating surgeon will be expected to seek the patient’s permission via the consent process as per *Montgomery*, summarized in four key points – all material risks disclosed, adequate time and space for the patient to consider their options, all reasonable treatment options discussed, and must not bombard the patient with excessive information. The importance of patient autonomy in consent in Western legal systems can be traced at least as far back as 1914, when Judge Cardozo stated “…every human being of adult years and sound mind has a right to determine what should be done with his body…”^27^ In acute trauma, it has to be accepted that time is limited for the patient to consider their options, with possibly just minutes to decide. Material risk is what the reasonable patient in that position would need to know (objective), and what risks in the doctor’s view the particular patient would consider important (subjective).^28^ This leaves two potential loose ends from *Montgomery* – what constitutes all reasonable treatment alternatives, and who decides what they are? Does the doctor or the patient filter the list of possible treatments, to end up with the list of reasonable treatments from which to choose? Snake oil and homeopathy may be alternative treatments, but can they be considered reasonable treatment alternatives? There has to be a filtering mechanism to produce the definitive list from which to choose.

*Montgomery* says: “…it is not possible to consider a particular medical procedure in isolation from its alternatives…There are choices to be made, arguments for and against each of the options to be considered, and sufficient information must be given so that this can be done…That is not necessarily to say that the doctors have to volunteer the pros and cons of each option in every case…[the patient] cannot force her doctor to offer treatment which [the doctor] considers futile or inappropriate…”

After a couple of relatively minor skirmishes in the lower courts in Scotland,^29,30^ matters came to a head in the case of *McCulloch*,^31^ decided in the Supreme Court in 2023. In a non-orthopaedic case – the use of non-steroidal anti-inflammatory drugs in pericarditis – the five justices reached a unanimous decision that narrowing down from ‘possible’ alternative treatments to ‘reasonable treatment alternatives’ is an exercise of clinical judgement, and therefore to be judged subjectively from the perspective of the doctor. The doctor, not the patient, filters the possible treatments to those that the doctor believes to be reasonable in the circumstances. This judgment is not without controversy, with published comment from both lawyers and surgeons, including two of the faculty members of the BOA’s ‘Law for Orthopaedic Surgeons: Avoiding Jeopardy’ course.^32^ In an online blog, barrister and Assistant Coroner Leila Benyounes lamented that while *Montgomery* had been a step away from ‘doctor knows best’ and towards protecting patients’ autonomy, surely *McCulloch* is regressively narrowing patient choice?^33^ Meanwhile, upper gastrointestinal and bariatric surgeon Abeezar Sarela posited that different surgeons having differing views on which treatments are reasonable for a given situation may lead to inequalities and injustice in the availability of reasonable treatments.^34^

## Conclusion

When considering ‘reasonable treatment alternatives’ in the consent process and under the jurisdiction of civil law, there are echoes from our earlier considerations of ‘proper medical treatment’ within the criminal law. While these two concepts arise from different origins within the law, there is considerable overlap between the two. As part of the satisfactory consent process, the surgeon must discuss all ‘reasonable treatment alternatives’, as determined by the *Bolam* test.^35^ To satisfy the criminal law and secure the medical exception, the surgeon must offer ‘proper medical treatment’, which is also determined by the *Bolam* test. In this way, surgeons are awarded the privilege to operate on their fellow humans, conferred by both patient and wider society. To operate on the injured patient is both a privilege and a huge responsibility. The privilege has been defined and refined over centuries of the development of surgery within society. The responsibility is not to be taken lightly, as people’s future health and wellbeing depend upon receiving proper medical treatment. Surgeons, both as individuals and in organized groups such as Specialty Associations and the Royal Colleges, must strive to uphold the privilege and responsibility to the highest standards.

**Take home message**

- Society has vested in surgeons the privilege to operate on the trauma patient, providing 'proper medical treatment', by means of the medical exception to the criminal law.

- This is linked to, but discrete from the fully informed, autonomous consent of the adult patient with capacity, which arises from the civil law.

- Surgeons are usually well aware of the latter criterion following Montgomery, but should also have some awareness of the former. This is the sum total of how orthopaedic trauma surgeons are allowed to do what we do.
